# Coral epigenetic responses to nutrient stress: Histone H2A.X phosphorylation dynamics and DNA methylation in the staghorn coral *Acropora cervicornis*

**DOI:** 10.1002/ece3.4678

**Published:** 2018-11-23

**Authors:** Javier A. Rodriguez‐Casariego, Mark C. Ladd, Andrew A. Shantz, Christian Lopes, Manjinder S. Cheema, Bohyun Kim, Steven B. Roberts, James W. Fourqurean, Juan Ausio, Deron E. Burkepile, Jose M. Eirin‐Lopez

**Affiliations:** ^1^ Environmental Epigenetics Laboratory, Institute of Water and Environment, Department of Biological Sciences Florida International University Miami Florida; ^2^ Department of Ecology, Evolution and Marine Biology University of California Santa Barbara California; ^3^ Seagrass Laboratory, Institute of Water and Environment, Department of Biological Sciences Florida International University Miami Florida; ^4^ Department of Biochemistry and Microbiology University of Victoria Victoria British Columbia Canada; ^5^ School of Aquatic and Fishery Science University of Washington Seattle Washington

**Keywords:** acclimatization, cnidarians, DNA methylation, global change, histones, pollution

## Abstract

Nutrient pollution and thermal stress constitute two of the main drivers of global change in the coastal oceans. While different studies have addressed the physiological effects and ecological consequences of these stressors in corals, the role of acquired modifications in the coral epigenome during acclimatory and adaptive responses remains unknown. The present work aims to address that gap by monitoring two types of epigenetic mechanisms, namely histone modifications and DNA methylation, during a 7‐week‐long experiment in which staghorn coral fragments (*Acropora cervicornis) *were exposed to nutrient stress (nitrogen, nitrogen + phosphorus) in the presence of thermal stress. The major conclusion of this experiment can be summarized by two main results: First, coral holobiont responses to the combined effects of nutrient enrichment and thermal stress involve the post‐translational phosphorylation of the histone variant H2A.X (involved in responses to DNA damage), as well as nonsignificant modifications in DNA methylation trends. Second, the reduction in H2A.X phosphorylation (and the subsequent potential impairment of DNA repair mechanisms) observed after prolonged coral exposure to nitrogen enrichment and thermal stress is consistent with the symbiont‐driven phosphorus limitation previously observed in corals subject to nitrogen enrichment. The alteration of this epigenetic mechanism could help to explain the synergistic effects of nutrient imbalance and thermal stress on coral fitness (i.e., increased bleaching and mortality) while supporting the positive effect of phosphorus addition to improving coral resilience to thermal stress. Overall, this work provides new insights into the role of epigenetic mechanisms during coral responses to global change, discussing future research directions and the potential benefits for improving restoration, management and conservation of coral reef ecosystems worldwide.

## 1 INTRODUCTION

Hermatypic (i.e., reef‐building, stony) corals constitute the structural basis of reef ecosystems, providing the foundation for over 25% of marine and coastal biodiversity. Unfortunately, during the last decades, coral reefs have experienced dramatic declines worldwide, caused by local and global anthropogenic stressors (Pandolfi et al., 2003). The sessile lifestyle and long lifespan of corals increase their vulnerability to a rapidly changing environment (Cunning & Baker, 2012; Nesa & Hidaka, 2009), but also support the idea that their evolutionary success relies on a remarkable level of phenotypic plasticity (Barshis et al., 2013; Bruno & Edmunds, 1997; Dimond & Roberts, 2016; Dixon, Bay, & Matz, 2014). Although a high degree of genotypic diversity can be found in some coral species (Ayre & Hughes, 2000, 2004 ; Souter, 2010), it is becoming increasingly clear that the plasticity provided by this mechanism will not be enough to keep up with the rapid progression to a warmer, more polluted, more acidic and carbonate‐limited ocean (Hoegh‐Guldberg et al., 2007; Hughes et al., 2017). Such a dark perspective has sparked the interest for the study of environmentally acquired nongenetic modifications (i.e., microbiome and epigenome dynamics) in these organisms, given their intrinsic potential to increase coral acclimatization and adaptation rates under rapidly changing environments (Palumbi, Barshis, Nikki, & Bay, 2014; van Oppen, Oliver, Putnam, & Gates, 2015). For instance, recent reports have revealed that specific symbiont strains can provide corals with higher tolerances to thermal stress (Leal et al., 2015; Silverstein, Cunning, & Baker, 2015, 2017 ), and that coral responses to different drivers of global climate change do in fact involve changes in the epigenome (i.e., DNA methylation) (Beal, Rodriguez‐Casariego, Rivera‐Casas, Suarez‐Ulloa, & Eirín‐López, 2018; Eirin‐Lopez, & Putnam, 2019; Liew et al., 2018; Putnam, Davidson, & Gates, 2016)

Organismal responses to environmental changes involve the activation of different mechanisms operating at diverse levels, from early genetic responses (Hoffmann & Willi, 2008) to whole‐individual physiological responses (Boyd et al., 2015; Shultz, Zuckerman, Stewart, & Suski, 2014). While different, all these mechanisms invariably require the modulation of the expression of specific sets of genes, promoting dynamic and sometimes reversible responses facilitating the onset of acclimatized phenotypes (Stillman & Armstrong, 2015). Epigenetic modifications, defined as phenomena and mechanisms that cause heritable (both mitotically and/or meiotically) chromosome‐bound changes to gene expression, not involving changes to DNA sequence (sensu Deans & Maggert, 2015), are at the center of this regulatory process (Eirin‐Lopez, & Putnam, 2019). Among the different epigenetic mechanisms known so far, DNA methylation is the most studied in all types of organisms (Schübeler, 2015), including corals where recent studies have characterized DNA methylation levels in the germ line and evidenced the involvement of this mechanism in responses to ocean acidification (Dimond & Roberts, 2016; Dixon et al., 2014; Liew et al., 2018; Marsh, Hoadley, & Warner, 2016; Putnam et al., 2016). Yet, studies elucidating the links between DNA methylation and gene expression, the interaction among different types of epigenetic mechanisms, as well as their precise involvement in responses to different drivers of global climate change in ecologically and environmentally relevant organisms, are still lacking (Beal et al., 2018).

Among the multiple threats posed by global change, anthropogenic nutrient pollution constitutes one the major drivers of coral decline (Fabricius, 2005; Wagner, Kramer, & van Woesik, 2010; Wooldridge, 2009). Their potential effects include increased coral bleaching (Cunning & Baker, 2012; Vega Thurber et al., 2014; Wooldridge, 2009), disease (Zaneveld, McMinds, & Thurber, 2017), reduced growth rates (Dunn, Sammarco, & LaFleur, 2012; Shantz & Burkepile, 2014), and impaired reproduction (Loya, Lubinevsky, Rosenfeld, & Kramarsky‐Winter, 2004). A possible mechanism underlying these deleterious effects is the rapid proliferation of symbiont populations triggered by the disruption of the nitrogen (N)‐limited environment maintained by the coral host inside the symbiosome (Downs et al., 2002; Nesa, Baird, Harii, Yakovleva, & Hidaka, 2012). The resulting phosphorus (P) starvation damages the photosynthetic machinery and alters the ionic balance in the symbiont thylakoid membranes (Pogoreutz et al., 2017; Wiedenmann et al.., 2012), subsequently increasing the export of reactive oxygen species (ROS) to the intracellular space while intensifying oxidative and DNA damage in both the host and the symbiont (Baruch, Avishai, & Rabinowitz, 2005; Ezzat, Maguer, Grover, & Ferrier‐Pagès, 2016; McGinty, Pieczonka, & Mydlarz, 2012; Nesa et al., 2012; Saragosti, Tchernov, Katsir, & Shaked, 2010; Wiedenmann et al., 2012). Overall, the effects of nutrient pollution will work synergistically with other stressors (particularly thermal stress) increasing bleaching at a mechanistic level (Pogoreutz et al., 2017) and coral mortality (Nesa & Hidaka, 2009; Yakovleva et al., 2009).

Although the potential ways in which nutrient and thermal stress can affect corals are well studied (Brown, 1997; D'Angelo & Wiedenmann, 2014; Nielsen, Petrou, & Gates, 2018), the identity and the precise role of the epigenetic mechanisms linked to acclimatory and adaptive responses to these stressors remain unknown. In order to fill that gap, the present work conducted a field experiment consisting of two different types of coral nutrient enrichments (treatment 1, nitrogen only; treatment 2, nitrogen + phosphorus) using the staghorn coral *Acropora cervicornis* as model organism. Given that a thermal stress event was observed in the study are at the same time that this experiment was taking place, the obtained results provide a unique opportunity to analyze the synergies between both types of stress mediating epigenetic responses in field conditions. Two types of epigenetic mechanisms were studied for that purpose, including histone modifications [histone H2A.X phosphorylation also known as gamma‐H2A.X, a histone modification involved in DNA repair and a universal marker of DNA damage (González‐Romero et al., 2012; Maré Chal & Zou, 2013)] and DNA methylation. It is hypothesized that nutrient enrichment will accelerate the growth of the symbiont population within the holobiont, resulting in a higher production of ROS which will in turn cause DNA damage, triggering an increase in gamma‐H2A.X (associated to DNA repair activation) and changes in DNA methylation. It is also hypothesized that gamma‐H2A.X formation will be impaired in corals exposed only to N enrichment (treatment 1), due to the P limitation caused by proliferation of symbionts in the absence of a P supply. Consequently, corals subject to N enrichment (treatment 1) would be expected to experience lower levels of DNA repair, encompassing deleterious phenotypic effects.

## 2 METHODS

### 2.1 Study site, experimental and sampling design

**Figure 1:**
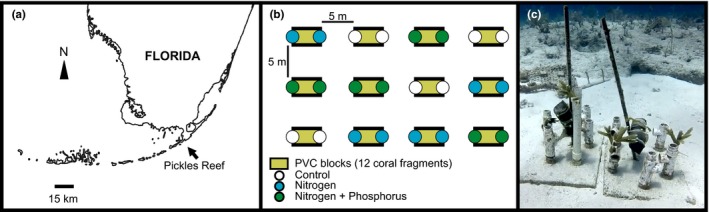
(a) Field experiment site location in Pickles Reef, Upper Florida Keys, Key Largo, FL (25°00′05″ N, 80°24′55″W). (b) Nutrient exposure experiment design consisting of 12 blocks evenly distributed across the study area (*n* = 4 blocks per treatment), randomly assigned to one of three treatment conditions: control (c), Nitrogen enrichment (N), and Nitrogen and Phosphorous enrichment (N + P). (c) Each coral fragment was secured to PVC tubing set in a base of concrete using nylon cable ties, for a total of 12 fragments per block

Nutrient exposures were conducted using a common garden experiment in a large sand flat located near Pickles Reef in the Upper Florida Keys, Key Largo, FL (Figure 1a) (25°00′05″N, 80°24′55″W) in approximately 5–7 m depth of water. Ambient nutrient conditions are relatively oligotrophic at this site (dissolved inorganic nitrogen [DIN] < 1.2 µM, soluble reactive phosphorus [SRP] < 0.04 µM; Zaneveld et al., 2016), making it a suitable location to test the effects of nutrient enrichment on corals. A total of 144 fragments of the staghorn coral *A. cervicornis* (three parental colonies, 7–13 cm in length) were obtained from a nearby offshore coral nursery operated by the Coral Restoration Foundation (permit no: FKNMS 2014‐071). Each coral fragment was secured to a 50 cm tall section of PVC tubing (4 cm diameter) set in a base of concrete using nylon cable ties, for a total of 12 fragments per stand (Figure 1b,c). Twelve experimental stands were distributed in a randomized block design across the study area with ≥2 m separation between them. Each stand (*n* = 4 per treatment) was randomly assigned to one of three treatment conditions as follows: Control (Ctrl), nitrogen enrichment (N), and nitrogen + phosphorous enrichment (N + P). Controls were replicated in the same way treatments were to account for the potential environmental variability typical of field experiments. Coral fragments attached to stands were allowed to acclimate for more than 10 days without treatment until any visible wounds resulting from the fragmentation process healed. N enrichment was achieved using Florikan 0‐19‐0 slow release ammonium nitrate fertilizer (300 g) as detailed by (Vega Thurber et al., 2014); N + P enrichment was obtained by combining 0‐19‐0 slow release ammonium nitrate fertilizer (300 g) with 80 g of 40‐0‐0 slow release Super phosphate fertilizer. Ctrl stands were not exposed to any nutrient source. In both N and N + P treatments, nutrient exposure was achieved through the diffusion of nutrients in water by evenly dividing the fertilizer into two perforated PVC tubes, wrapped in mesh and secured at opposing sides of each block via cable ties. This method was previously validated to triplicate the ambient levels of DIN and SRP for a period of 30–45 days in similar conditions (Heck, Pennock, Valentine, Coen, & Sklenar, 2000; Sotka & Hay, 2009; Vega Thurber et al., 2014).

Epigenetic modifications in invertebrates can occur rapidly after exposure to environmental stress (Gonzalez‐Romero et al., 2017; Rivera‐Casas et al., 2017; Suarez‐Ulloa, Gonzalez‐Romero, & Eirin‐Lopez, 2015). Therefore, coral fragments were sampled at three different times during day 1 of exposure (1, 2, and 5 hr), day 2, day 7, and weekly thereafter for the next 4 weeks. For each sample, one coral fragment was randomly collected from each stand (*n* = 4 coral fragments per treatment, *n* = 12 fragments per sampling). Fragments were collected by cutting the cable ties securing them to the stands and were subsequently stored in individual sealed sterile plastic bags. Once all samples were collected, bags were transported to the surface and immediately flash frozen in liquid nitrogen. Fragments were divided into sub‐samples for nutrient analyses and for molecular analyses, finally stored at −80°C.

### 2.2 Nutrient quantification

N and P content were quantified in tissue from coral fragments collected during the experiment, including increased sampling frequency during week 1. This sampling design is consistent with the findings of Achituv, Ben‐Zion, and Mizrahi (1994) and Muller‐Parker, Cook, and D’Elia (1994), suggesting that the most significant nutrient changes in coral tissue occur within that period. Coral holobiont (the unit formed by the coral animal and its associated microorganisms consisting of bacteria, archaea, fungi, viruses, and protists including Symbiodiniaceae dinoflagellate algae) tissue was removed from a portion of each of the fragments sampled using an airbrush loaded with ultrapure water and was dried to a constant weight at 60°C and homogenized to powder. Samples were subsequently fumed with HCl for 14 days to completely remove the skeletal inorganic carbon fraction (Szmant, Ferrer, & FitzGerald, 1990) and dried at 70°C until no further weight change was observed. Carbon (C) and N content were measured in aliquots (10 mg) of dried and decalcified tissues using a FISONS elemental analyzer (NA1500, Loughborough, UK). P content was analyzed sensu Solórzano and Sharp, (1980) using a modification adapted for tissue (Fourqurean, Zieman, & Powell, 1992). Briefly, 5–10 mg of dried tissue were placed into glass scintillation vials, diluted with 0.5 ml of 0.17 M Na_2_SO_4_ and 2 ml of 0.017 M MgSO_4_, and dried again at 90°C. The resulting powder was incubated at 500°C for 3 hr and cooled down to room temperature. A total of 5 ml of 0.2 N HCl was added to these oxidized and dried samples and incubated at 80°C for 30 min, after which they were diluted with 10 ml of deionized water and allowed to stand overnight for the insoluble ash to settle. The phosphate concentration in the solution was determined as SRP using a colorimetric assay. The elemental content was calculated on a percentage of dry weight basis, and elemental ratios were calculated on a mole: mole basis. Data were collected following time frames reported in the literature, greater than or equal to 10 days (Godinot, Houlbrèque, Grover, & Ferrier‐Pagès, 2011) but less than 8 weeks (Godinot, Ferrier‐Pagès, & Grover, 2009), while considering the rapid initial changes accounted in the sampling design (Achituv et al., 1994; Muller‐Parker, Cook, et al., 1994; Muller‐Parker, Cook, et al., 1994). Accordingly, samples for the first 3 days were used as initial time (*T*_1_) and then organized into samples greater than 10 days but less than 8 weeks (*T*_2_ and *T*_3_) to ensure nutrient uptake representation.

### 2.3 Symbiont density analysis

The density of coral symbiont (Symbiodiniaceae) algae was quantified across treatments and exposure times by removing all tissue from the coral skeleton using the procedure detailed above. Upon extraction, tissue samples were homogenized using a tissue grinder and centrifuged for 5 min using a hand centrifuge to isolate symbiont cells. Each sample was subsequently divided into five technical replicates (100–300 μl each) and symbiont cells were quantified using a hemocytometer (Weber Scientific, Hamilton, NJ) in an inverted microscope (Leica, Buffalo Grove, IL). The extracted fragment's surface area (cm^2^) was estimated using the aluminum foil method (J. Marsh, 1970). Quantifications were averaged across technical replicates to produce mean symbiont density (cells × cm^‐2^) for each fragment. To determine whether enrichments impacted *Symbiodinium *growth rates, we tested for differences in the *Symbiodinium *density through time within each of the three treatments. To do so, we used linear mixed effects models with hours since enrichment began as a continuous predictor and included growth platform as a random factor to account for nonindependence within the platforms (using *χ* ^2^ with 1 *df* to test whether symbiodinium growth rate significantly differs from zero through time). Tests were conducted using the nlme package in R (Pinheiro, Bates, DebRoy, Sarkar, & R Core Team, 2018). Normality and homogeneity of variance were confirmed via quantile–quantile plots and plots of fitted versus residual values.

### 2.4 Histone Isolation, separation, and detection

Histone proteins re isolated as described elsewhere and adapted to coral tissue in the present work (Rivera‐Casas et al., 2017). Accordingly, 5 mg of holobiont tissue were homogenized in a buffer consisting of 100 mM KCl, 50 mM Tris‐HCl, 1 Mm MgCl_2%,_ and 0.5% Triton X‐100 (pH 7.5) and containing a protease inhibitor mixture. After homogenization and incubation on ice for 5 min, samples were centrifuged at 12,000 *g* for 10 min at 4°C. The resulting pellets were re‐suspended in 0.6 N HCl, homogenized, and centrifuged again. The supernatant extracts were precipitated with six volumes of acetone at −20°C overnight and centrifuged at 12,000 *g* for 10 min at 4 ºC. The acetone pellets were dried using a Vacufuge concentrator (Eppendorf, Hamburg, Germany) and stored at −80°C. Histone protein separation was carried out in SDS‐PAGE gels using ClearPAGE SDS gels 4%–20% (C.B.S. Scientific, Del Mar, CA). Gels were stained with 0.2% (w/v) Coomassie blue in 25% (v/v) 2‐propanol, 10% (v/v) acetic acid and de‐stained in 10% (v/v) 2‐propanol, 10% (v/v) acetic acid. Additional histone separation was carried out using high‐performance liquid chromatography (HPLC) as described in Rivera‐Casas et al. (2017). Histone proteins were detected using commercial antibodies in western blot analyses, including anti‐H2A.X (H2A.X.ab, Abcam Cambridge, MA; H2A.Xry; Raybiotech, Norcross, GA) and anti‐γH2A.X (γ‐H2A.X ab, Rockland, Pottstown, PA; γ‐H2A.Xry, Raybiotech). SDS‐PAGE gels were electro‐transferred to a nitrocellulose membrane (C.B.S. Scientific) and processed as described elsewhere (Rivera‐Casas et al., 2017). Membranes were incubated with a secondary goat anti‐rabbit antibody (Rockland) that was subsequently detected using enhanced chemiluminescence (Amershan ECL Prime Western Blotting Detection Reagent; GE Healthcare Life Sciences, Piscataway, NJ). Results were analyzed using the ChemiDoc‐It TS2 Imager image analysis system (UVP Inc., San Gabriel, CA).

### 2.5 RNA extraction, cDNA synthesis, and qPCR reactions

Total RNA was extracted from coral holobiont tissue using Ribozol Reagent (Amresco, Solon, OH), and digested with PerfeCTa DNase I (Quanta Biosciences, Gaithersburg, MD) to eliminate residual genomic DNA. cDNA was synthesized using qScript cDNA Supermix (Quanta Biosciences), and expression analyses were subsequently performed by means of quantitative PCR (qPCR). Primers specific for H2A.X and H4 histone genes were designed based on sequences retrieved from GenBank databases for *A. cervicornis* and *A. formosa* (Table 1) using the Primer‐BLAST software (Ye et al., 2012). Histone H4 was used for normalization purposes. Primer efficiencies were calculated based on the slope of calibration curves constructed using 10‐fold dilution steps, according to the formula *E* = 10^−1/slope^. The resulting gene expression profiles were subsequently examined in *A. cervicornis* RNA samples by measuring SYBR green incorporation in a LightCycler 96 System (Roche, Mannheim, Germany). cDNA amplifications were carried out in 45 cycles under the following conditions: Pre‐incubation at 95°C for 10 min, denaturalization at 95°C for 10 s, annealing at 60°C for 10 s, and elongation at 72°C for 10 s, including a final melting gradient up to 97°C using a ramp of 4.4°C × s^‐1^ to confirm primer specificity. Each individual reaction was carried out in triplicate, including negative controls (no template control, NTC; non‐reverse transcription control, NRTC). Results were recorded as normalized ratio values by the LightCycler 96 Software version 1.1 following the Pfaffl method (Pfaffl, 2001).

**Table 1:** qPCR primers used in histone gene expression analyses and species used as references for their design

Gene	Primer name	Sequence (5′→3′)	Species
H2A.X	Ac‐H2A.X‐Fw	CTCAGGGAGGTGTTTTGCCA	*Acropora cervicornis*
	Ac‐H2A.X‐Rv	TGGCTTTGGGATGATTTCCCT	
H4	Af‐H4‐Fw	CCGGGCTCCCAGTAAAATGT	*Acropora formosa*
	Af‐H4‐Rv	TGTCGTATGGGGGAGGGATT	

### 2.6 gamma‐H2A.X/H2A.X ratio analysis

The quantification of histone H2A.X and its phosphorylated form (gamma‐H2A.X) was implemented in coral samples from different experimental treatments by using a commercial ELISA kit (Raybiotech), providing a simultaneous semi‐quantitative measure of the gamma‐H2A.X/H2A.X ratio in a single experiment. For that purpose, 10 mg of coral tissue from each of three samples per treatment per time were solubilized in 500 µl of commercial lysis buffer and incubated on ice for 30 min. After centrifugation (18,000 *g* for 10 min at 4°C), 100 µl of each lysate were loaded by duplicate in anti‐H2A.X precoated microplate along with positive and negative controls provided in the kit, and samples were incubated overnight at 4°C. Subsequently, 100 µl of detection antibodies (anti‐H2A.X [S139] or anti‐pan‐H2A.X), Horseradish Peroxidase (HRP)‐conjugated anti‐rabbit IgG (against secondary antibodies), and TMB One‐Step Substrate Reagent were added to the plate following manufacturer's indications. The TMB substrate was incubated for 30 min in the dark with shaking, and 50 µl of Stop Solution were added to each well before reading absorbances in a ELx808IU microplate reader (Biotek, Winooski, VT) at 450 nm.

### 2.7 DNA extraction and DNA methylation analysis

Genomic DNA was purified as described elsewhere and adapted to coral tissue in the present work. Briefly, tissue homogenates were incubated at 50°C for 2 hr with CTAB lysis buffer (100 mM Tris, 20 mM EDTA, 1.2 M NaCl, 2% CTAB, pH 8.0) and proteinase K, completing DNA extraction following the phenol–chloroform protocol (Sambrook & Russell, 2006). DNA methylation was quantified in genomic DNA samples by measuring the amount of 5‐methyl‐Cytosines (5‐mC), using the MethylFlash Global DNA Methylation (5‐mC) ELISA kit (Epigentek, Farmingdale, NY). Accordingly, three genomic DNA samples per treatment/time were loaded in duplicate to ELISA plates, along with positive (polynucleotide with 50% of 5‐mC) and negative controls (polynucleotide with 50% of unmethylated Cytosine), all with binding solution. All samples were diluted to a final concentration of 9.645 ng/µl in NanoPure water, corresponding to 77.12 ng of DNA in each well. Once binding was completed, 100 µl of capture antibody, detection antibody, developer solution, and stop solution were sequentially added, performing intercalated incubations and plate washes, following manufacturer indications. The absorbance (OD) resulting from the colorimetric reaction was quantified at 450 nm in a ELx808IU microplate reader (Biotek). Quantification of 5‐methyl‐Cytosine content (ng) was performed following the calculations suggested by the manufacturer.

### 2.8 Statistical analyses

All results are presented as mean values of replicate samples ±standard error, unless indicated otherwise. All statistical analyses were performed with respect to controls to separate the contributions of the experimental variables. The statistical significance of the effect of blocks, treatments, and exposure time was evaluated by means of Two‐Way ANOVA and One‐Way ANOVA when required. This approach was appropriate for the analysis of P content, histone H2A.X quantification, and DNA methylation after transformation to natural logarithm. In all cases, data were confirmed to follow a normal distribution (Shapiro–Wilk Test, *p* > 0.05) and variance homogeneity (Brown‐Forsythe Test, *p* > 0.05). The analysis of N content data (including N:P molar ratios) was done by means of a Two‐Way PERMANOVA with Euclidian distance using 9,999 permutations (Anderson, 2001). Although this is primarily a multivariate method, it performs as a univariate test (equivalent to ANOVA) under the current experimental data conditions, avoiding the assumption of normality (Anderson, 2017) and allowing for the analysis of interactive effects (Doropoulos et al., 2014). PERMDISP was used to test for homogeneity of dispersion (equivalent to homoscedasticity). Post‐hoc Tukey‐HSD tests and the Holm‐Sidak method were used for multiple comparisons when appropriate. All analyses were carried out using R 3.4.1 (R Core Team, 2017), except the Two‐Way PERMANOVA that was performed using PAST 3.18 (Hammer, Harper, & Ryan, 2001) and the PERMDISP analysis that was performed with Primer v6 (Clarke & Gorley, 2006)

## 3 RESULTS

### 3.1 Nutrient quantification and thermal monitoring during experimental treatments

**Figure 2:**
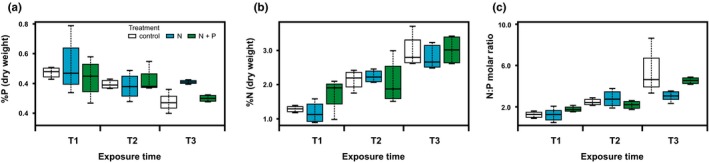
(a) Nutrient content in tissue from staghorn coral fragments exposed to the different enrichment treatments implemented in the present work. (a) Phosphorus tissue content in coral fragments expressed as percent of dry mass of reactive phosphate; (b) Nitrogen tissue content in coral fragments expressed as percent of dry mass; (c) N:P molar ratio. Exposure times are defined as follows: *T*_1_, hour 1 to day 3; *T*_2_, day 3 to 20; and *T*_3_, day 20 to 35

The nutrient enrichment treatments implemented in the present work did not cause coral mortality, and no bleaching or disease was evident during the experiment. In addition, it was determined that the studied parameters were not influenced by the block design (*p* > 0.05, Table 2). N and P levels in the holobiont displayed particularly low values (Figure 2, Table 3), with %P around 0.4% and %N around 2.1 for all treatments. Nonetheless, while neither N or P content displayed significant differences among treatments (%P: *F*_(2,34) _= 0.744, *p = *0.483; %N: *F*_(2,34)_ = 0.692 *p* = 0.427), both parameters showed significant changes during the span of the experiment, decreasing in the case of P content (*F*_(4,34) _= 5.960, *p = *0.007, Figure 2a), and increasing in the case of N content (*F*_(4,34) _= 10.527, *p < *0.001, Figure 2b). As a result, N:P molar ratios displayed a significant dependence with time (*F*_(4,34)_ = 13.62, *p* < 0.001; Figure 2c), as well as a statistical dependence with the nutrient enrichment treatments assayed (*F*_(2,34)_ = 1.8245 *p* = 0.05). Interestingly, although tissue nutrient analyses were not very sensitive to the nutrient addition treatments developed on the reef, results showed an antagonistic response of N and P through time, evidencing a mild nutrient enrichment in holobiont tissues.

**Table 2:** Two‐way ANOVA analysis of the contribution of block design to the studied variables. %P and %N represent percentage of dry weight for each element

Variable	Source of variation	*df*	*F*	*p*
%P	Block	3	0.574	0.636
	Treatment × block	6	0.495	0.808
%N	Block	3	1.167	0.336
	Treatment × block	6	0.407	0.869
gamma‐H2A.X/H2A.X	Block	3	1.128	0.345
	Treatment × block	6	0.183	0.833
DNA methylation	Block	3	1.920	0.156
	Treatment × block	6	0.883	0.419

**Table 3:** Nutrient content in corals (holobiont) exposed to control (C), enriched nitrogen (N), and enriched nitrogen and phosphorus (N + P) treatments

Treatment	%P	%N	%C	N:P	C:N
C	0.382 (0.096)	2.136 (0.790)	15.592 (5.049)	3.011 (2.163)	6.350 (0.498)
N	0.420 (0.148)	2.135 (0.755)	15.298 (4.665)	2.875 (2.310)	6.286 (0.731)
N + P	0.404 (0.106)	2.116 (0.715)	15.791 (4.615)	2.493 (1.132)	6.480 (0.556)

Note

Values represent mean and standard deviation (in parentheses) for all samples collected during a 4‐week‐long exposure (*n* = 24). N:P and C:N represent molar nitrogen:phosphorus and carbon:nitrogen ratios, respectively. %P, %N and %C represent percentage of dry weight for each element.

**Figure 3:**
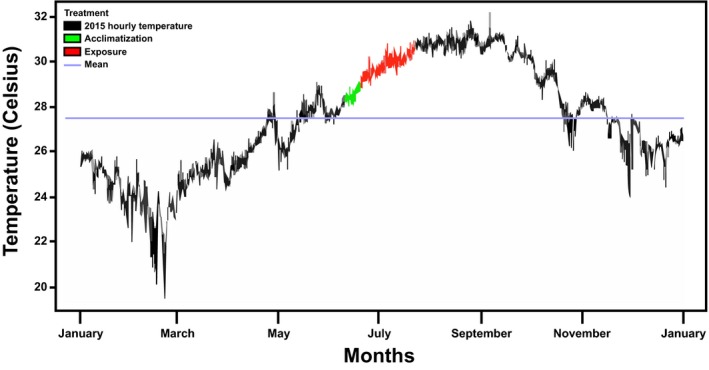
Hourly water column temperatures in the Florida Keys National Marine Sanctuary, site 225, for the year 2015. The blue line represents the mean value for the temperature registered in this station for the year. The periods corresponding to the different stages of the experiment are indicated in green (acclimatization of coral fragments) and red (exposure of coral fragments to nutrient enrichment treatments)

Given that the present experiment was directly developed in the reef, factors other than nutrient exposure could be affecting the observed results, notably fluctuating thermal regimes. Consequently, temperature data corresponding to the experimental site (long‐term monitoring station, 4 km away and at similar depth, site 225, 25°00.807', 80°22.677') were subsequently analyzed to evaluate this possibility (Figure 3). Results revealed a temperature increase in the lower portion of the water column (up to 40 cm from the bottom) from 28.39 ± 0.15°C at the beginning of acclimatization period, to 30.52 ± 0.05°C by the end of the experiment. This represents a net increase of more than 2°C during the exposure period, reaching the bleaching threshold reported for *A. cervicornis* in the Florida Keys (30.5°C; Manzello, Berkelmans, & Hendee, 2007). Based on this observation, the effect of thermal stress was added to that of nutrient stress, in order to better evaluate their combined effect on coral epigenetic responses.

### 3.2 Changes in symbiont population densities across nutrient treatments

Symbiont density analyses revealed a significant increase in the symbiont populations of *A. cervicornis* corals subject to nutrient enrichment treatments during the course of the present experiment (Table 4), as compared with the constant density levels observed in corals subject to control conditions. Additionally, the obtained results revealed that changes in symbiont densities were significantly influenced by the specific nature of the nutrient treatments as follows: on one hand, corals exposed to N only enrichment (treatment 1) displayed a twofold increase respect to control corals; on the other, a fourfold increase was observed in corals exposed to N + P enrichment (treatment 2). Along with nutrient quantification analyses, these results further support the efficiency of the nutrient exposures developed during the present work.

**Table 4:** Mixed effects models analysis of modifications in symbiont population densities in *A. cervicornins* during the course of the present experiment under control (C), enriched nitrogen (N), and enriched nitrogen and phosphorus (N + P) treatments^a^

Treatment	*χ* ^2^	Slope ± *SE*	*p*
C	2.184	0.00034 ± 0.00023	0.140
N	4.400	0.00084 ± 0.00040	0.036
N + P	14.061	0.00100 ± 0.00028	<0.001

a The slope represents the linear estimate of how the symbiont population changes through time (10^6^ cell × hour^‐1^) in the different treatments. See Statistical Methods in the Methods section of this work for additional details on symbiont density analyses.

### 3.3 Changes in histone H2A.X phosphorylation during nutrient and thermal stress

**Figure 4:**
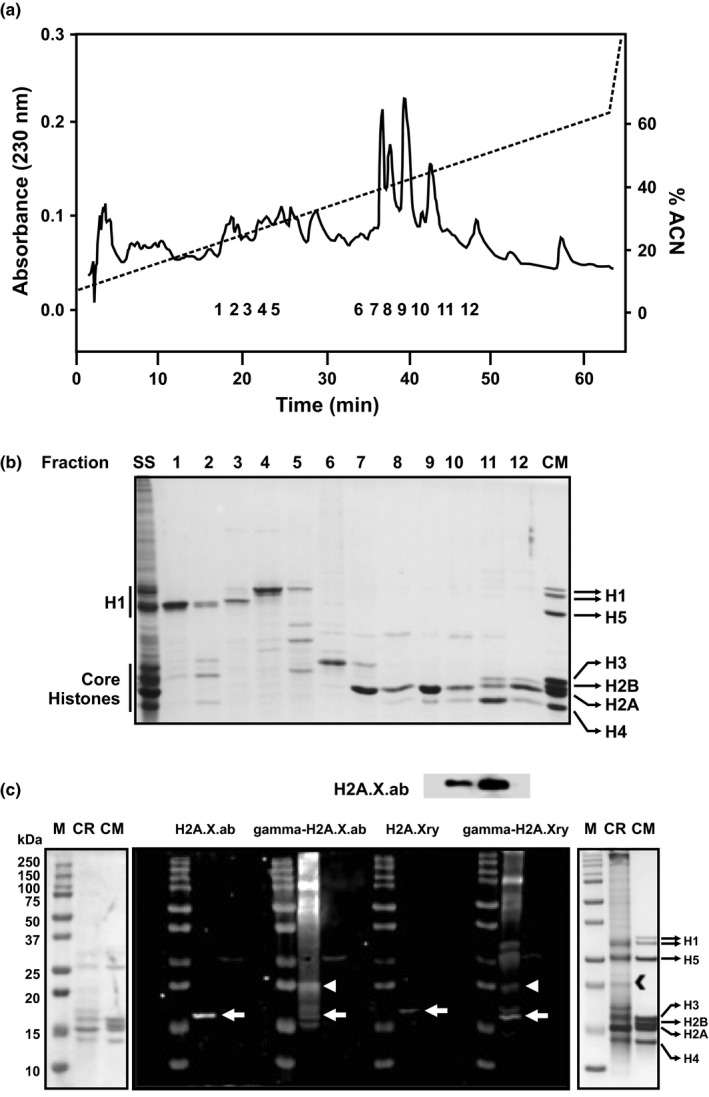
(a) Purification profile of acid‐extracted staghorn coral histones across an acetonitrile gradient (ACN) using HPLC. The analyzed histone fractions are indicated by numbers 1–12. (b) SDS‐PAGE separation of HPLC histone fractions 1–12 revealing linker and core histones, as well as diverse histone‐like proteins present in the coral holobiont. (c) Western blot immunodetection of histone variant H2A.X and its phosphorylated form (gamma‐H2A.X) to validate antibody specificity (above) and of HCl‐extracted histones from *Acropora cervicornis* (below) using commercial antibodies H2A.X.ab (Abcam), γ‐H2A.X ab (Abcam), H2A.Xry (RayBiotech), and γ‐H2A.Xry (RayBiotech). ACN, acetonitrile; CM, chicken marker; M: molecular weight marker; CR, coral tissue extraction; SS, starting sample

**Figure 5:**
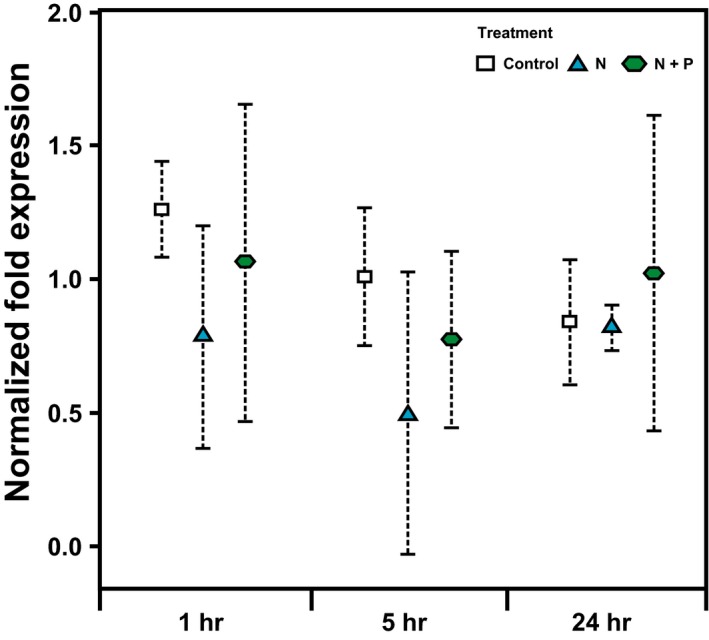
Histone H2A.X gene expression levels in staghorn coral during the first 24 hr of exposure to different nutrient treatments. Plots represent mean normalized ratios in relation to the study calibrator (Histone H4) ± *SE* (*n* = 2)

Histones from *A. cervicornis* were extracted, isolated, and purified for the first time in the present work, including different fractions containing linker and core histones, as well as diverse histone‐like proteins present in the coral holobiont (Figure 4a,b). In addition, H2A.X and its phosphorylated form gamma‐H2A.X were immunodetected using western blot analyses (Figure 4c), validating the use of different commercial antibodies for their detection in corals. The role of H2A.X during coral responses to nutrient and thermal stress was studied at two different functional levels. First, coral H2A.X gene expression patterns were analyzed using coral‐specific qPCR primers specifically designed using *A. cervicornis* and *A. formosa* sequences retrieved from GenBank databases as references (Table 1). The obtained results revealed homogeneous gene expression levels across the different nutrient treatments during the first 24 hr of exposure (*F*_(2,9)_ = 1.569, *p* = 0.265, Figure 5, Supporting information Figure S1), suggesting that the main role of coral H2A.X during responses to nutrient stress (temperature was not high enough to cause stress during the first 24 hr) does not take place at the transcriptional level.

**Figure 6:**
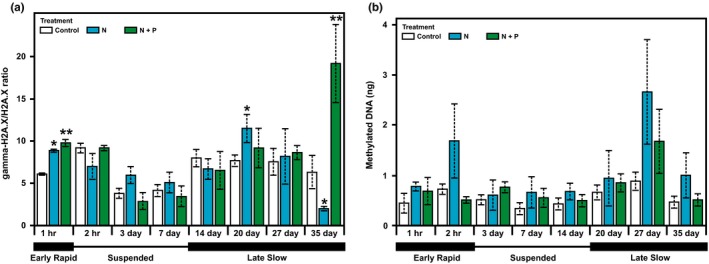
(a) Characterization of histone H2A.X phosphorylation levels in staghorn coral fragments across different nutrient treatments, estimated as the ratio between phosphorylated H2A.X (gamma‐H2A.X) and its nonmodified form (H2A.X). Each plot represents mean ± *SE* (*n* = 3). The level of significance of the post‐hoc Holm‐Sidak test is indicated as *p < 0.05, **p < 0.01. The response was divided into three parts: early rapid response (hour 1), suspended response (hour 2–day 7), and late slow response (after day 7). b. Characterization of global DNA methylation levels in staghorn coral fragments across different nutrient treatments, estimated as total mass of methylated (5‐methyl‐Cytosine) DNA. Each plot represents mean ± *SE* (*n* = 3, biological replicates). The response was divided into three parts mirroring gamma‐H2A.X, defined as follows: early rapid response (hour 1–2), suspended response (hour 2–day 14), and late slow response until the end of the experiment (after day 14).

The analysis of the epigenetic effects mediated by H2A.X was subsequently expanded to the post‐translational level, based on the well‐established link between H2A.X phosphorylation and DNA damage repair. For that purpose, gamma‐H2A.X levels were quantified during coral exposure to different nutrient treatments under increasing temperature, revealing significant differences between different treatments at specific sampling times (*F*_(14,40)_ = 4.361, *p* < 0.001) in spite of the high variability in the response of the controls. These results can be interpreted as indicative of DNA damage occurring in higher rates under enriched conditions, based on the stress marker nature of the gamma‐H2A.X modification. Accordingly, the observed response can be divided into three major stages (Figure 6a): first, an early rapid response consisting of a significant increase in gamma‐H2A.X was observed during the first hour in corals subject to both N and N + P treatments (Tukey‐HSD test, *q* = 16.264, *p* = 0.003); second, a suspended gamma‐H2A.X response was observed in both treatments starting from hour 2 to day 7; and third, a late slow response in gamma‐H2A.X over a longer period of time that was observed after day 7. In this last period, phosphorylation reached significantly different values in both enrichment treatments as follows: on one hand, gamma‐H2A.X became significantly greater than controls after a 20‐day exposure to the N treatment (Tukey‐HSD test, *q* = 4.734, *p* = 0.036) and after a 35‐day exposure to N + P treatment (Holm‐Sidak test, *t* = 4.057, *p* < 0.001); on the other, a reduction in gamma‐H2A.X levels was observed in coral fragments subject to N enrichment for more than 20 days, displaying significant differences respect to controls upon reaching the 35‐day mark (Holm‐Sidak test,* t* = 2.394, *p* = 0.021).

### 3.4 Changes in DNA methylation during nutrient and thermal stress

In addition to histone modifications, the role of DNA methylation during coral responses to nutrient stress was analyzed in the present work to account for the potential interaction among multiple mechanisms during epigenetic effects in response to environmental stress. In the present case, however, DNA methylation analyses did not detect significant differences among different nutrient treatments (*F*_(2,44)_ = 2.505, *p* = 0.093) or across different time points (*F*_(7,44)_ = 2.081, *p* = 0.066) (Figure 6b). Nonetheless, the obtained results evidenced that the mean DNA methylation content in corals exposed to N enrichment was twice as much as that experienced by control corals at hour 1, hour 2, day 7, day 27, and day 35. The same was observed for corals exposed to N + P for day 27. Interestingly, this trend is similar (although no significant correlation was observed) to that observed for gamma‐H2A.X (Figure 6a), including an initial rapid response, followed by a suspended response and by a late slow response lasting until the end of the experiment.

## 4 DISCUSSION

The present work constitutes one of the few pioneering efforts investigating the role of epigenetic mechanisms during environmental responses in corals, more precisely to nutrient and thermal stress. In doing so, this work also expands recent efforts combining the study of multiple epigenetic mechanisms during environmental epigenetic responses in marine invertebrates, including histone variants (and their modifications) and DNA methylation (Gonzalez‐Romero et al., 2017; Li et al., 2018). The obtained results constitute the first description of the histone variant H2A.X and its phosphorylated form, gamma‐H2A.X, in a stony coral species. Such findings, together with the histone diversity previously described in cnidarians (Reddy, Ubhe, Sirwani, Lohokare, & Galande, 2017; Török et al., 2016) as well as in Symbiodiniaceae dinoflagellates (Lin et al., 2015), unveil the potential contribution that chromatin‐associated proteins convey during epigenetic effects and inheritance linked to environmental epigenetic responses in this group (Beal et al., 2018). Along with the study of DNA methylation levels, this work starts shaping our knowledge about the potential interactions among different epigenetic mechanisms mediating environmental responses, as well as their modulation by the combined action of different stressors (e.g., nutrients and temperature).

### 4.1 Coral nutrient content does not predict environmental nutrient exposure

Nutrient quantification analyses revealed a lack of correlation between nutrient content in the coral holobiont and the expected environmental nutrient levels derived from the experimental exposures. Nonetheless, a nutrient enrichment effect was evidenced by the N:P molar ratios estimated during exposures (Figure 2c), as well as by the increase in symbiont population densities across treatments (Table 4). Although nutrient content in water was not evaluated in this work, studies using the same enrichment strategy in the same location and season, successfully enriched the water column by approximately 3 µM N and 0.3 µM P in a 1 m radius around nutrient diffusers (Vega Thurber et al., 2014; Zaneveld et al., 2016), supporting the success of the present experimental approach in locally elevating nutrient concentrations available to experimental coral fragments. Indeed, it has been demonstrated that changes in environmental nutrient concentrations are not necessarily linked to changes in tissue content (Achituv et al., 1994; Godinot et al., 2009; Godinot, Ferrier‐Pagès, Montagna, & Grover, 2011; Muller‐Parker, Cook, et al., 1994; Muller‐Parker, Cook, et al., 1994; Muller‐Parker, Mccloskey, Hoegh‐Guldberg, & Mcauley, 1994). Accordingly, multiyear nutrient enrichment experiment (including both N and P) demonstrated a strong nutrient stoichiometric homeostasis and high constancy in coral holobiont tissue, regardless of elevated external nutrient levels, and even in the presence of a significant increase in the ^15^N isotope in corals exposed to N enrichment (Koop et al., 2001). Consequently, based on these observations as well as on the results obtained in the present work, the lack of a cause‐effect relationship between environmental nutrient enrichment and the nutrient levels determined for coral tissues could be due to a rapid nutrient turnover in the holobiont.

On the other hand, nutrient content changed significantly with time and independently of nutrient treatment, suggesting that other factors may be influencing nutrient content in coral tissue. Among the different environmental parameters chiefly affecting coral fitness, it is well known that thermal stress can modify coral nutrient uptake ratios (Ezzat et al., 2016; Godinot, Houlbrèque, et al., 2011) and regulate phosphate transfer to symbiotic vacuoles (Miller & Yellowlees, 1989). The analysis of thermal regimes during the present experiment revealed a progressive increase in water temperature in the area of study (Figure 3), potentially affecting the observed nutrient dynamics. Accordingly, among the different reports addressing the effect of thermal stress on nutrient uptake ratios, at least one has described a sharp increase in N uptake (with no change in P intake) in corals subject to mild thermal stress (29°C, Godinot, Ferrier‐Pagès, et al., 2011; Godinot, Houlbrèque, et al., 2011), matching the observations described in the present work (Figure 2a,b). On the other hand, alternative studies have described an inverse pattern in coral species subject to severe thermal stress (>30°C, Ezzat et al., 2016; Godinot, Ferrier‐Pagès, et al., 2011; Godinot, Houlbrèque, et al., 2011). Altogether, these results are illustrative of the complexity of nutrient stress responses in corals, being possible that the thermal variation experienced by experimental corals (28–30°C) contributed to the observed trends in nutrient contents.

### 4.2 gamma‐H2A.X participates in coral epigenetic responses to nutrient and thermal stress

Coral exposure to elevated nutrient levels can promote the rapid proliferation of symbionts, leading to a potential increase in the production and export of ROS (Cunning & Baker, 2012; Ezzat et al., 2016; Marubini & Davies, 1996; Nesa & Hidaka, 2009; Wiedenmann et al., 2012; Wooldridge, 2009), as well as in DNA damage (Lesser, 2006). Under conditions of nutrient imbalance and/or thermal stress, such deleterious effects are likely to be exacerbated by the damage experienced by the photosynthetic machinery (Pogoreutz et al., 2017), as well as by the disruption of the symbiont's membrane composition (Wiedenmann et al., 2012). Given the well‐established role of histone H2A.X and its phosphorylated form during the activation of DNA repair mechanisms in eukaryotes (Maré Chal & Zou, 2013; Suarez‐Ulloa et al., 2015), the modifications observed in gamma‐H2A.X/H2A.X levels are consistent with the role of this mechanism mediating epigenetic effects during coral responses to nutrient stress, supporting the link between exposure to nutrient/thermal stress and the presence of DNA damage.

The results from gene expression analyses indicate that the role played by H2A.X does not appear to take place at a transcriptional level (Figure 5, Supporting information Figure S1). Only two other studies have evaluated H2A.X gene expression in marine invertebrates, with contradictory results. On one hand, increased H2A.X.1 and H2A.X.2 mRNA levels were found in *Hydra* exposed to the genotoxic agent bleomycin (Reddy et al., 2017). On the other, no expression changes were observed on variants H2A.X, H2A.Z, and macroH2A during the exposure of the Eastern oyster *Crassostrea virginica* to marine toxins (Gonzalez‐Romero et al., 2017). Nonetheless, both studies reported increased gamma‐H2A.X levels upon exposure to environmental stress (Gonzalez‐Romero et al., 2017; Reddy et al., 2017), supporting that the main functional role of this variant during DNA repair is regulated at a post‐translational level.

The results obtained in this work suggest a link between environmental (nutrient/thermal) stress and histone H2A.X phosphorylation in corals. However, the observed patterns were complex. First, basal gamma‐H2A.X levels (gamma‐H2A.X/H2A.X ratio >3) in corals are higher than those found in other eukaryotes including humans (Ji et al., 2017) and marine invertebrates (Gonzalez‐Romero et al., 2017). Such peculiarity can be interpreted in the context of the recurrent state of hyperoxia to which corals are subject during the day, resulting from the photosynthetic activity of symbiotic algae (Kuhl, Cohen, Dalsgaard, Jorgensen, & Revsbech, 1995; Shashar, Cohen, & Loya, 1993). This includes the production of ROS (Dykens, Shick, Benoit, Buettner, & Winston, 1992), requiring frequent mitigation of the subsequent oxidative damage in the coral holobiont (Richier, Furla, Plantivaux, Merle, & Allemand, 2005; Roth, 2014). Precisely, such complex interaction between the coral host and the algal symbiont could also explain the high variability observed for gamma‐H2A.X/H2A.X ratios in controls. Second, the transition from early rapid response, to suspended response, to late slow response periods in gamma‐H2A.X levels (Figure 6a) agrees with coral acclimatory responses, necessary to activate molecular and physiological mechanisms temporally restoring homeostasis until additional responses (usually more intense and persistent than the previous) are required. Indeed, a similar dynamic response was observed during coral exposure to thermal stress, involving two pulses in the expression of the heat shock protein *hsp70* linked to acclimatization periods to different levels of stress (Gates & Edmunds, 1999). Similarly, Moya, Ganot, Furla, and Sabourault (2012) observed a rapid and transient transcriptomic response to stress in the anemone *Anemonia viridis*, followed by a second response after 5 or 21 days depending on the combination of thermal stress and UV exposure. The obtained results are further supported by the identification of pulse‐like or transient responses in the expression and activity of stress proteins in coral larvae (Rodriguez‐Lanetty, Harii, & Hoegh‐Guldberg, 2009; Voolstra et al., 2009), as well as in mollusks exposed to thermal stress (Anestis, Lazou, Portner, & Michaelidis, 2007).

The final stage of the experiment was particularly interesting regarding histone H2A.X dynamics, as gamma‐H2A.X levels displayed significant differences with respect to controls but with different signs depending on the nutrient treatment. Accordingly, gamma‐H2A.X levels increased drastically in corals exposed to N + P by day 35, which not only agree with a prolonged exposure to nutrient stress, but also with the increment in water temperature (more than 2°C at this point). On the contrary, gamma‐H2A.X levels decreased significantly by day 35 in corals exposed to N only enrichment, which is a remarkable observation considering that these individuals were also subject to thermal stress (and therefore require as much DNA repair as possible). This is probably one of the most interesting results in the present work, as it provides support for the hypothesis suggesting that N enrichment will promote P starvation in the coral holobiont (Wiedenmann et al., 2012), hampering the phosphorylation of H2A.X and subsequent activation of DNA repair mechanisms. In addition, P starvation has been proposed to increase thermally driven damage to photosystem II (Pogoreutz et al., 2017), as well as to limit the capacity of the thylakoid membrane to contain ROS (Wiedenmann et al., 2012), further exacerbating DNA damage in cells where DNA damage repair (by way of gamma‐H2A.X formation) is already seriously impaired. On the other hand, a higher level of H2A.X phosphorylation (indicative of DNA damage sensing and repair) will be expected in corals exposed to N + P treatment after 35 days, as corroborated by the obtained results, thanks to the presence of P as part of that treatment, therefore preventing the harmful effects of P starvation.

Overall, the consequences of the impairment in H2A.X phosphorylation are enormous, as these will directly affect the ability of the coral holobiont to activate DNA damage repair mechanisms (Albino et al., 2009). Indeed, the alteration of this epigenetic mechanism could help explaining the synergistic effects of nutrient imbalance and thermal stress on coral fitness, increasing bleaching and mortality (Ezzat et al., 2016; Wooldridge, 2009). Similarly, these results also support the positive effect of P addition in order to improve coral resilience to thermal stress (Ezzat et al., 2016).

### 4.3 Global DNA methylation

Among the different epigenetic mechanisms known to date, DNA methylation is the best studied in marine organisms (Beal et al., 2018; Eirin‐Lopez, & Putnam, 2019). In the present work, the analysis of global DNA methylation did not detect significant differences among different nutrient treatments or across different time points (Figure 6b). Such result is surprising, based on the multiple reports describing changes in DNA methylation levels in marine organisms subject to different environmental stimuli (Beal et al., 2018; Eirin‐Lopez, & Putnam, 2019). A possible explanation could involve the scale at which DNA methylation was quantified in the present work. Accordingly, DNA methylation was estimated at a global genomic level which provides little resolution; therefore, the marginal lack of significance observed could result from limited replication. In addition, DNA methylation was quantified for the coral holobiont (including both the coral host and the algal symbiont) introducing another potential source of variability affecting the results obtained. In addition, the canceling effect that specific local modifications may have on each other cannot be neglected. Lastly, both promoter and gene‐body methylation (or the lack thereof) appear to contribute to phenotypic plasticity in marine invertebrates (Eirin‐Lopez, & Putnam, 2019; Gavery & Roberts, 2013; Li et al., 2018; Marsh & Pasqualone, 2014), making the study of this epigenetic mechanism extremely complex in this group. An illustration of such complexity is exemplified by responses to stress involving an increase in DNA methylation at specific genomic regions accompanied by demethylation at others, resulting in a net genome‐wide DNA methylation level similar to that present in controls (same number of DNA methylation marks but at different genomic regions). Despite the limitations of the method, the contribution of DNA methylation to coral stress responses is hinted by the trends observed, including pulsed changes in DNA methylation mirroring those observed in the case of gamma‐H2A.X/H2A.X ratios.

Since pulsed responses would facilitate immediate responses upon stress exposure, followed by the activation of other complementary mechanisms mediating longer‐term responses, it would not be surprising if DNA methylation also follows such trend by regulating the expression of genes linked to other mechanisms involved in the maintenance of genome integrity. Further analyses addressing changes in DNA methylation variation at higher resolution (i.e., single nucleotide level) will be necessary in order to clarify that aspect.

## 5 CONCLUSIONS

This work constitutes a pioneering effort describing coral epigenetic modifications during responses to nutrient and thermal stress, including histone modifications and DNA methylation. The obtained results support the presence of the specialized histone variant H2A.X and its phosphorylated form (gamma‐H2A.X) in stony corals. The relationship between gamma‐H2A.X levels and coral exposure to stress appears to be consistent with the role of this histone modification activating DNA repair responses. Such function is further supported by the observed impairment of gamma‐H2A.X formation after prolonged exposure to N enrichment, underscoring the detrimental effects that P limitation bears on the epigenetic mechanisms preserving coral genome integrity. Although the observed modifications in DNA methylation during nutrient and thermal stress were not large enough to be statistically significant, the contribution of this epigenetic mechanism to coral stress responses should not be disregarded based on the followings: (a) the global nature of the DNA methylation estimations developed in this work; (b) the similarity between the shape of DNA methylation trends (two major pulses during the experiment), and that of the gamma‐H2A.X response observed over the course of exposures; and (c) the complexity of DNA methylation responses to environmental stress described in marine invertebrates. Overall, this effort constitutes a first step toward understanding the intricacies of the mechanisms regulating environmental epigenetic responses in marine organisms. Further efforts will be required to bring this research to the next level, including genome‐wide, single‐nucleotide resolution level studies to elucidate the regulatory relationships between different epigenetic mechanisms and the genes involved in acclimatory and adaptive responses. Similarly, the study of the interaction between the genome and the epigenome will help understand how population diversity shapes epigenetic responses in marine populations, along with the implications for the implementation of epigenetic selection methods. Although these goals will be even more challenging in the specific case of corals (given the contribution of the symbiont genome and epigenome to the phenotype of the holobiont), the potential benefits for improving restoration, management, and conservation of coral reef ecosystems worldwide clearly justify that effort.

## AUTHORS CONTRIBUTION

JR‐C designed the study, developed experiments, analyzed the data, and wrote the manuscript; MCL performed fieldwork experiments and data analysis; AS performed fieldwork experiments and data analysis; CL developed experiments; MC developed experiments; BK developed experiments; SR developed experiments; JF developed experiments, and analyzed the data; JA developed experiments, and analyzed the data; DB designed the study, provided materials, and wrote the manuscript; JME‐L designed the study, provided materials, performed fieldwork, developed experiments, analyzed the data, and wrote the manuscript.

## DATA ACCESSIBILITY

Data corresponding to sampling locations, nutrient data, climate data, and epigenetic analyses on which the present research is based, are deposited in the repository of the CREST‐CAChE NSF Center at Florida International University and publicly available at https://github.com/eelabfiu/acropora. Data will be made available to the public upon acceptance of the present work.
