# Supplementary figures and images for: Coral epigenetic responses to nutrient stress: Histone H2A.X phosphorylation dynamics and DNA methylation in the staghorn coral Acropora cervicornis
Source: Ecol Evol. 2018 Nov 23;8(23):12193–207. doi: 10.1002/ece3.4678 (PMC6303763; doi:10.1002/ece3.4678)

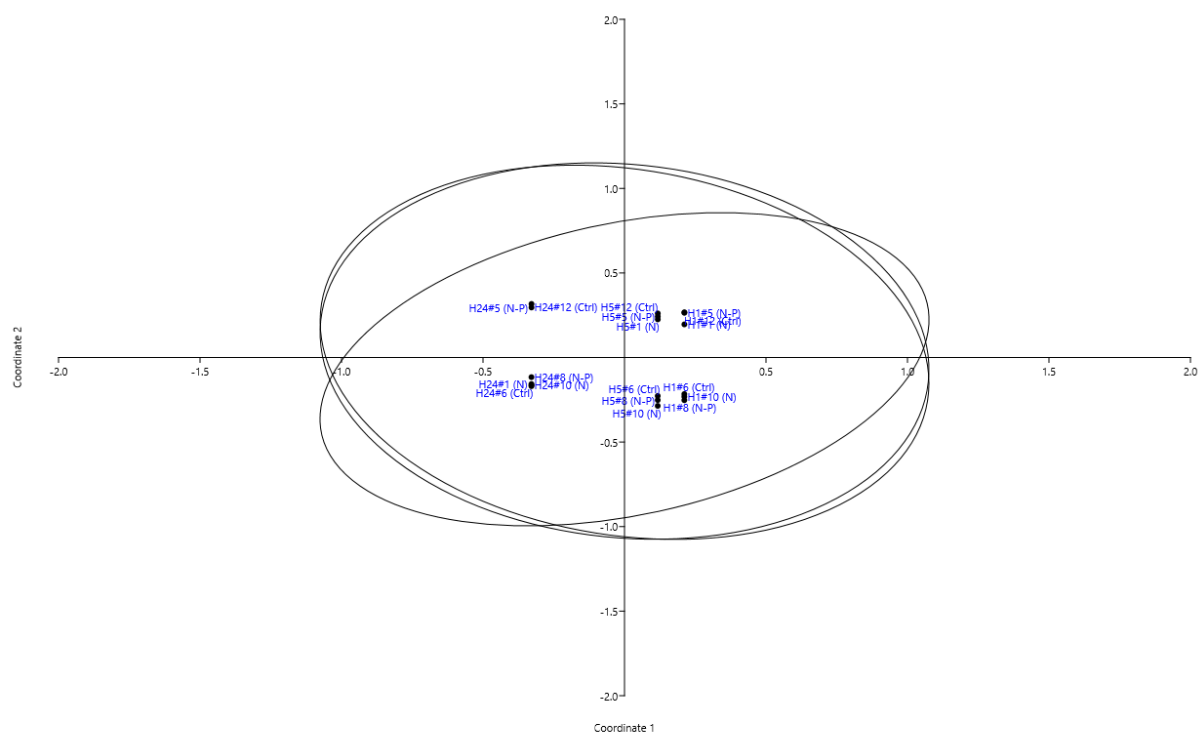

**Suppl. Fig. 1, Rodriguez-Casariago et al. 2018**

Supplement: Supplementary file 1 [file ECE3-8-12193-s001.pdf]
